# Comparative Effectiveness of Diabetes Self-Care Interventions in African-American Adults: A Three-Arm Randomized Controlled Trial

**DOI:** 10.1007/s11606-025-09882-z

**Published:** 2025-10-20

**Authors:** James E. Bailey, Satya Surbhi, Justin Gatwood, Susan W. Butterworth, Mace Coday, Ming Chen, Mary Lou Gutierrez, Sohul A. Shuvo, Ian M. Brooks, Bonnie L. Binkley, Carrie Jo Riordan, Helmut O. Steinberg, Cardella L. Leak, William R. Breen, Stanley W. Dowell, Elizabeth A. Tolley

**Affiliations:** 1 Tennessee Population Health Consortium, University of Tennessee Health Science Center, 956 Court Ave., Coleman D222, Memphis, TN 38163 USA; 2 Center for Health System Improvement, College of Medicine, University of Tennessee Health Science Center, Memphis, TN USA; 3 Division of General Internal Medicine, Department of Medicine, University of Tennessee Health Science Center, Memphis, TN USA; 4 Department of Preventive Medicine, University of Tennessee Health Science Center, Memphis, USA; 5 College of Pharmacy, University of Tennessee Health Science Center, Memphis, TN USA; 6 Institute for Health Outcomes and Policy, College of Graduate Health Sciences, University of Tennessee Health Science Center, Memphis, TN USA; 7 Department of Biomedical Informatics, University of Colorado Anschutz Medical Center, Aurora, CO USA; 8 Division of Endocrinology, Department of Medicine, University of Tennessee Health Science Center, Memphis, TN USA; 9 Honeycomb Management Services, Memphis, TN USA; 10 Methodist Le Bonheur Healthcare, Memphis, TN USA

## Abstract

**Background:**

Although studies have demonstrated that many patient-centered behavioral interventions are effective in improving diabetes self-care, little is known regarding their comparative effectiveness.

**Objective:**

The Management of Diabetes in Everyday Life (MODEL) Study sought to compare the effectiveness of promising alternative patient-centered behavioral interventions, including educational materials (EM) alone, health coaching (HC)+EM, and text messaging (TM)+EM, for improving diabetes self-care.

**Design:**

Three-arm pragmatic randomized controlled trial (ClinicalTrials.gov NCT02957513).

**Participants:**

A total of 666 African-American adults with uncontrolled diabetes (hemoglobin A1c [HbA1c] ≥ 8%) and multiple chronic conditions from 19 Mid-South US primary care practices randomly assigned to EM, HC+EM, or TM+EM in a 1:2:2 ratio.

**Interventions:**

EM-alone participants received culturally tailored and patient-vetted diabetes EM. HC+EM participants received motivational interviewing–based coaching from certified lay health coaches. TM+EM participants received personalized and tailored messages.

**Main Measures:**

Primary outcomes included patient-reported healthy eating, exercise, and medication adherence self-care behaviors. Secondary outcomes included HbA1c, body weight, quality of life, and primary care engagement.

**Key Results:**

All three arms significantly improved healthy eating, exercise, and HbA1c from baseline to 12 months. EM participants increased healthy eating days by 0.67 ± 0.19 day/week (95% CI, 0.30–1.04), HC+EM by 0.99 ± 0.15 day/week (95% CI, 0.70–1.28), and TM+EM by 1.36 ± 0.15 day/week (95% CI, 1.07–1.64). Improvements in exercise behaviors were similar in all arms. TM+EM participants experienced significantly greater improvement in healthy eating than EM-alone participants (0.69 ± 0.24 day/week). Average reduction in HbA1c was 0.76% ± 0.12% (p ≤ 0.0001) and 90 of 349 participants (25.8%) with complete data for HbA1c achieved reductions below 8%.

**Conclusion:**

The MODEL Study demonstrates that EM-alone, HC+EM, and TM+EM are effective for improving self-care behaviors and HbA1c. TM+EM is more effective than EM-alone in improving healthy eating. Low-cost EM, HC+EM, and TM+EM should be made more readily available in primary care settings nationwide.

**Graphical Abstract:**

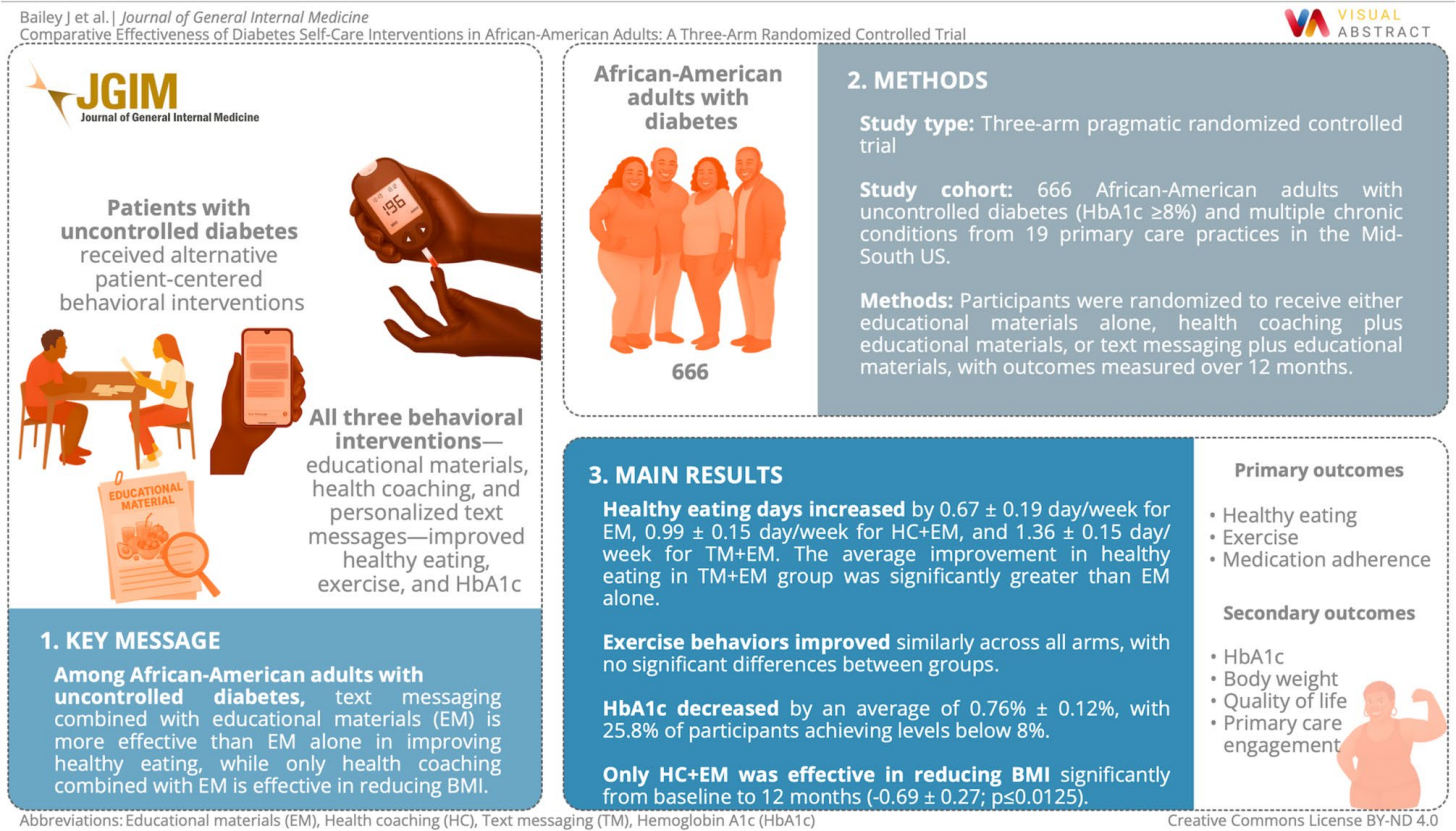

**Supplementary Information:**

The online version contains supplementary material available at 10.1007/s11606-025-09882-z.

Chronic diseases cluster in individuals, particularly in those with diabetes. Of the estimated 37 million US adults (14.7%) living with diabetes^1^, over 80% live with multiple chronic conditions (MCC)^2^. Patients with both uncontrolled diabetes and MCC are more likely to be hospitalized, experience complications, and die^3^. Uncontrolled diabetes is particularly common among African-American people with MCC in low-income and health professional shortage areas (HPSA)^4,5^. Limited access to primary care, poor primary care infrastructure, and poor patient engagement in diabetes self-care in HPSA lead to disproportionate suffering and death in African-American communities^3,6^. As a result, Black or African-American adults with diabetes are four times more likely than Whites to be hospitalized for uncontrolled diabetes, 2.3 times more likely to experience lower extremity amputation from diabetes, and 40% more likely to die from diabetes^7^.

Numerous systematic reviews and metanalyses have identified educational materials (EM), motivational interviewing–based health coaching (HC) and tailored text messaging (TM) as among the most promising and lowest-cost patient-centered strategies for expanding primary care capacity to support improved diabetes self-care^8–12^. Culturally tailored EM have longstanding evidence of effectiveness from pilot studies for improving diabetes self-care^8,13^, but are poorly delivered in real-world settings^14^.

Strong evidence from patient-centered outcomes research (PCOR) demonstrates the effectiveness of HC^9,15–18^ and TM^19–23^in comparison with usual care for improving diabetes self-care. The largest randomized controlled trials (RCTs) of HC for diabetes self-care all employed usual care controls and recruited from a single city, limiting generalizability^15–18^. Likewise, two major RCTs assessed the effectiveness of TM for diabetes self-care prior to the MODEL Study beginning in 2016^19,23^, but both employed usual care controls, neither compared TM to other active comparators such as HC and EM, and both lacked broad generalizability to primary care settings^19,23^. The two large RCTs assessing TM since 2016 also employed usual care as their primary comparator^21,22^. Although one of these recent RCTs also employed an active comparator control, it did not directly compare EM, TM, and HC provided for similar intensity and duration^21^. Unfortunately, none of these trials reported actual costs of studied interventions or cost-effectiveness, limiting their practical application and implementation potential. In summary, previous studies of HC and TM employed heterogeneous interventions, most employed usual care rather than the active comparators, and none reported actual costs or cost-effectiveness. Thus, the direct evidence needed to guide policy makers regarding the true comparative effectiveness of EM, TM, and HC in real-world settings is limited.

Our preliminary work documents a critical need to determine the diabetes self-care interventions most likely to succeed in reversing disparities in diabetes control and outcomes experienced by people of color^24,25^. The Management of Diabetes in Everyday Life (MODEL) Study aimed to determine the comparative effectiveness of patient-centered TM versus HC versus patient-vetted educational materials (EM) alone for African-American adults with uncontrolled diabetes.

## METHODS

### Trial Design, Setting, and Oversight

The MODEL Study (ClinicalTrials.gov NCT02957513) was a pragmatic, randomized controlled, three-group trial conducted from November 2016 through December 2020. The setting was 19 practices serving patients from low-income areas and HPSA^26^. The study was intentionally designed as a pragmatic trial according to Pragmatic Explanatory Continuum Indicator Summary-2 tool criteria and implemented in real-world primary care settings (Supplemental Table 1)^27^.

**Table 1:** Baseline Characteristics of Recruited Patients (*N* = 666)

Characteristics	HC (*N* = 258)	TM (*N* = 253)	EM (*N* = 155)
**Age, *n* (%)**			
< 60 years	166 (64.3)	166 (64.3)	100 (64.5)
≥ 60 years	92 (34.7)	84 (33.2)	55 (35.5)
**Gender, *n* (%)**			
Female	181 (70.2)	172 (68.0)	94 (60.6)
Male	77 (29.8)	81 (32.0)	81 (32.0)
**Marital status, *n* (%)**			
Married or living with partner	72 (27.9)	68 (26.9)	56 (36.1)
Single/separated/divorced/widowed	186 (72.1)	185 (73.1)	99 (63.9)
**Residence in HPSA, *n* (%)**			
HPSA	221 (85.7)	216 (85.4)	126 (81.3)
Non-HPSA	37 (14.3)	37 (14.6)	29 (18.7)
**Education, *n* (%)**			
Grades 6 to 12	44 (17.1)	52 (20.5)	29 (18.7)
GED/high school diploma	65 (25.2)	69 (27.3)	44 (28.4)
Some college/associate/bachelors/graduate degree/other, *n* (%)	149 (57.7)	132 (52.2)	82 (52.9)
**Health literacy**^†^**, *n* (%)**			
Low health literacy	72 (27.9)	68 (26.9)	39 (25.2)
High health literacy	186 (72.1)	185 (73.1)	116 (74.8)
**Urban/suburban-rural status**^†^**, *n* (%)**			
Residence in urban areas	220 (85.3)	218 (86.2)	122 (78.7)
Residence in suburban/rural areas	38 (14.7)	35 (13.8)	35 (13.8)
**Comorbidities**^‡^**, *n* (%)**			
Hypertension	234 (90.7)	233 (92.1)	140 (90.3)
Asthma	42 (16.3)	36 (14.2)	22 (14.2)
Hyperlipidemia	191 (74.0)	199 (78.7)	199 (76.8)
Arthritis	115 (44.6)	93 (36.7)	61 (39.4)
Cancer	12 (4.6)	10 (3.9)	11 (7.1)
Stroke	30 (11.6)	26 (10.3)	17 (11.0)
Chronic obstructive pulmonary disease	15 (5.8)	12 (4.7)	8 (5.2)
Congestive heart failure	22 (8.5)	33 (13.0)	17 (11.0)
Coronary artery disease	13 (5.0)	23 (9.1)	12 (7.7)
Cardiac arrhythmias	14 (5.4)	29 (11.5)	11 (7.1)
Depression	69 (26.7)	61 (24.1)	31 (20.0)
Chronic kidney disease	22 (8.5)	30 (11.9)	18 (11.6)
Osteoporosis	8 (3.1)	7 (2.8)	9 (5.8)
**Number of comorbidities, *n* (%)**			
1 comorbidity	34 (13.2)	36 (14.2)	19 (12.3)
2–3 comorbidities	140 (54.3)	130 (51.4)	84 (54.2)
≥ 4 comorbidities	84 (32.5)	87 (34.4)	52 (33.6)
**Smart phone ownership**^§^**, *n* (%)**			
Patients with no smart phone	45 (17.5)	45 (17.8)	23 (14.8)
Patients with smart phone	212 (82.5)	208 (82.2)	132 (85.2)
**Psychosocial complexity**^∥^**, *n* (%)**			
Low psychosocial complexity	155 (60.1)	152 (60.1)	99 (63.9)
High psychosocial complexity	103 (39.9)	101 (39.9)	56 (36.1)
**Medical complexity**^¶^**, *n* (%)**			
No	193 (96.5)	198 (95.6)	110 (95.6)
Yes	7 (3.5)	9 (4.4)	5 (4.4)
**HbA1c, mean (SD)**	10.2 (1.9)	10.2 (1.8)	10.3 (1.8)
**BMI, mean (SD)**	35.7 (8.4)	35.5 (9.1)	35.6 (9.4)

BMI, body mass index; EM, educational materials; HbA1c, hemoglobin A1c; HC, health coaching; HPSA, health professional shortage area; TM, text messaging

^*^High vs low health literacy categories are based on Chew’s validated 1-question screening instrument

^†^Urban vs. suburban and rural categories are based on Schanke-Mahl classification

^‡^Each chronic condition was a binary variable, with 1 indicating the presence of the chronic condition and 0 indicating the absence of the chronic condition

^§^Missing data for 1 patient

^∥^High psychosocial complexity is defined as either a positive screen for depression (using the 9-item Patient Health Questionnaire), anxiety (using the Generalized Anxiety Disorder Assessment), substance abuse (using the National Institute on Drug Abuse Quick Screen and AUDIT), or housing instability (using the Homelessness Screening Clinical Reminder)

^¶^High medical complexity is defined according to Camden Coalition and SafeMed “high-utilizer” criteria of ≥ 2 hospital admissions or 1 inpatient admission and ≥ 2 prior emergency visits in the past 6 months before enrollment in the study. Of 666 participants, hospitalization and emergency department visit data were available for 522 patients

^**^Data on baseline BMI was available for 340 study participants (136 in HC+EC, 126 in TM+EC, and 78 in EC alone)

Patients from the participating practices were recruited using (1) direct referral by clinic personnel, (2) telephone outreach using a regional practice-based research network, (3) review of patient charts, and (4) recruitment materials. All patients provided informed consent. Trial data were collected using Research Electronic Data Capture (REDCap) tool hosted by the University of Tennessee Health Science Center (UTHSC). The MODEL Study protocol was approved by the review boards of UTHSC and participating health systems.

### Randomization and Blinding

Eligible patients randomly assigned in a 1:2:2 ratio using blocks of five to receive either EM-alone, HC+EM, or TM+EM. Study participants, primary care physicians, health coaches, and research assistants who collected outcome data were unblinded. However, allocation was unknown to clinic personnel who recorded clinical outcome measures in routine clinical practice.

### Patient and Trial Groups

The inclusion criteria were (1) self-identification as African American; (2) age ≥ 18 years; (3) uncontrolled diabetes in the last 6 months (HbA_1c_≥ 8); (4) diagnosis of one or more of 13 additional chronic health conditions (i.e., hypertension, congestive heart failure, coronary artery disease, cardiac arrhythmias, hyperlipidemia, stroke, arthritis, asthma, cancer, chronic kidney disease, chronic obstructive pulmonary disease, depression, and osteoporosis); (5) receiving care at a participating practice; (6) mobile phone with text and voicemail capabilities; and (7) completing a 2-week run-in period demonstrating responsiveness to texts and voicemail messages left by program staff^26^. Diagnoses and HbA_1c_ were assessed using electronic health record (EHR) data.

Exclusion criteria were (1) inability to understand consent procedures; (2) inability to communicate in English; (3) pregnancy; (4) diagnosis of an unstable psychiatric condition, dementia, neurological disorder, or history of severe head trauma, brain tumor, or severe depression; (5) evidence of cognitive impairment; (6) uncontrolled psychiatric symptoms and/or behaviors; and (7) unwillingness or inability to participate as perceived by a study research assistant^26^.

### Interventions

**Figure 1:**
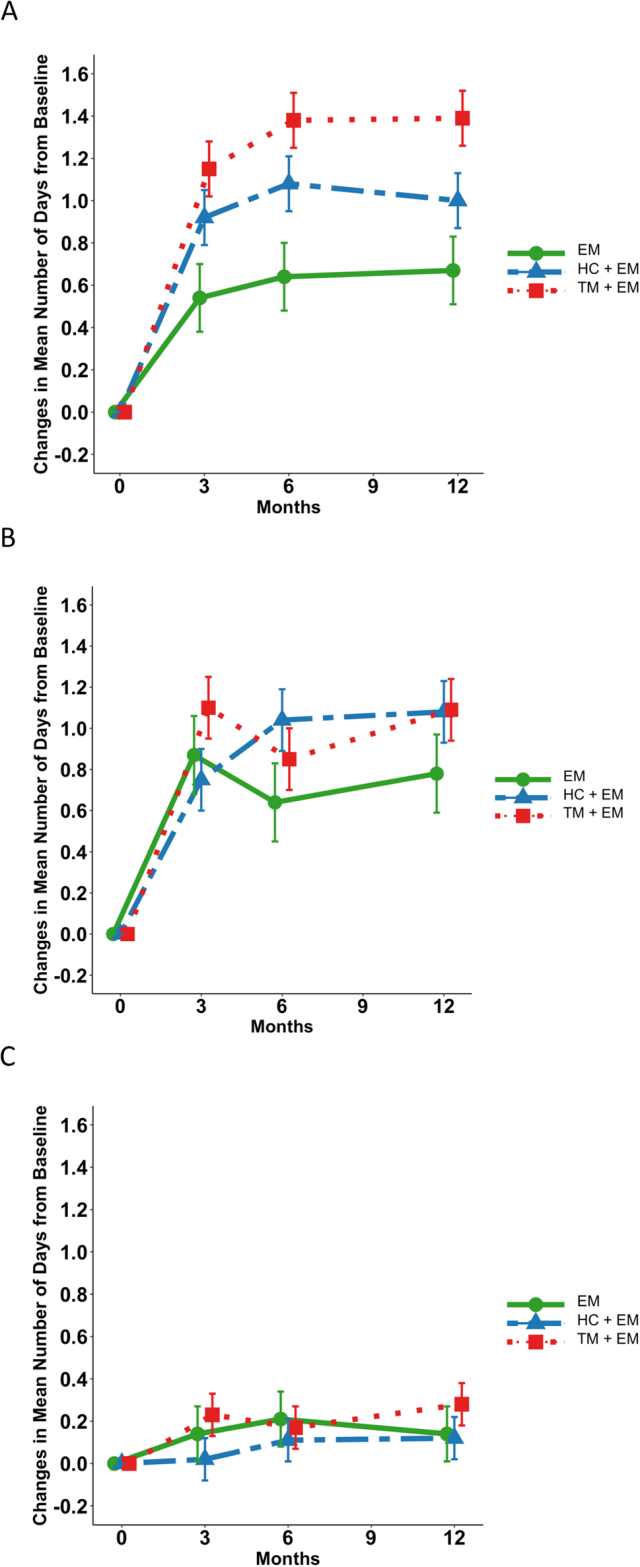
Mean change in patient-reported diabetes self-care activities among three study arms. **A** Change in healthy eating. **B** Change in exercise. **C** Change in medication adherence.

**Figure 2:**
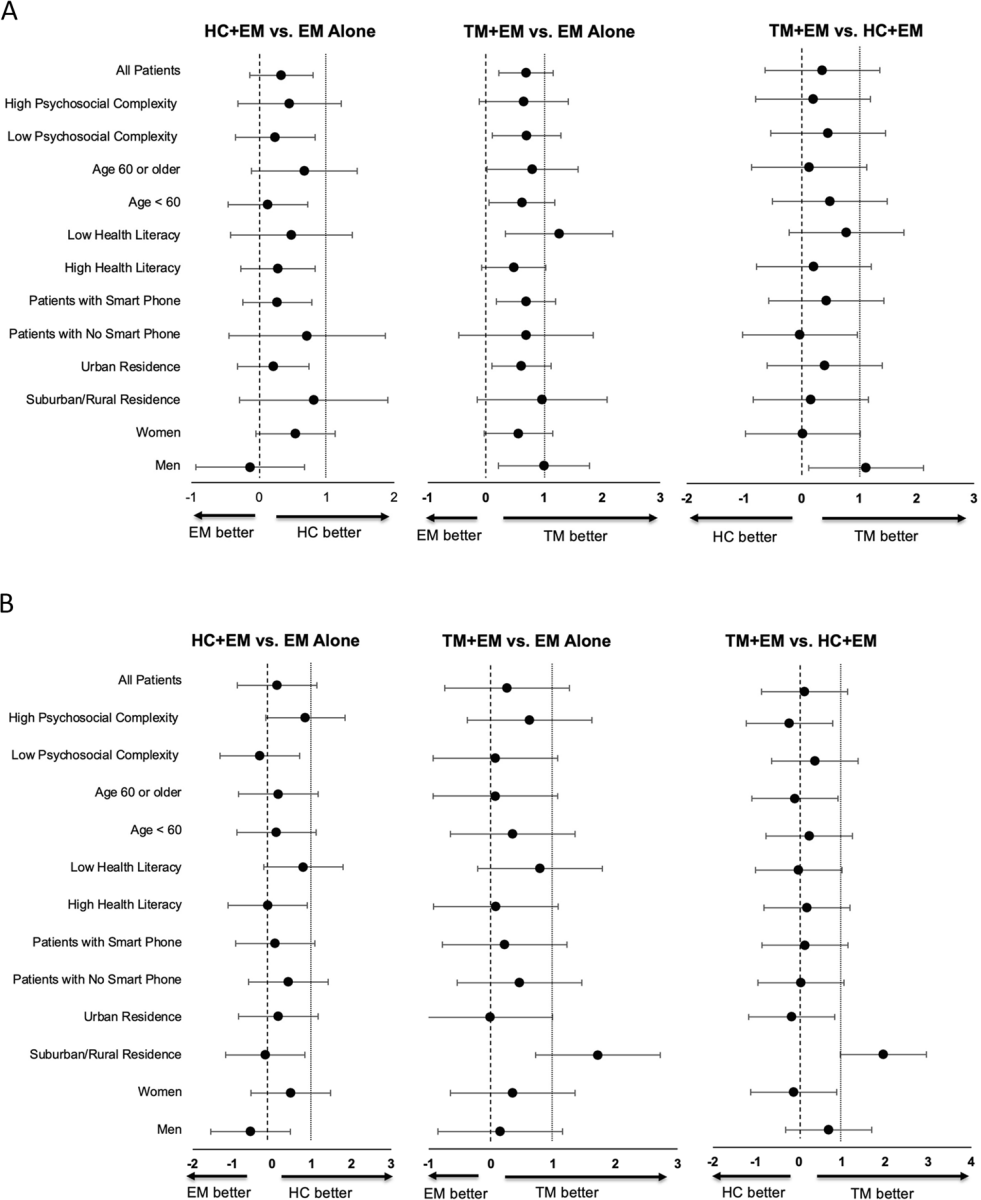
Subgroup analysis of treatment effects of educational materials (EM) alone, health coaching (HC+EM), and text messaging (TM+EM). **A** Healthy eating. **B** Exercise. The bars represent 95% confidence intervals for the comparative effectiveness within each of the indicated subgroups. Observed differences among subpopulations were not significant after adjusting for multiplicity.

The three MODEL Study intervention arms have been previously described^26^. All three arms focused on improving self-care activities related to general healthy eating, exercise, and medication adherence during a 1-year follow-up. All participants received study interventions in the context of routine care consistent with best practices for a pragmatic trial. The EM-alone arm served as an active comparator, which featured patient-vetted, high-quality print diabetes education materials and mobile phone-accessible online resources selected through a multistage process informed by best practices in the development and selection of diabetes educational materials^28,29^ to ensure cultural appropriateness for the target population (Supplement Table 2 and Figs. 1 and 2). This arm also received other enhanced usual care components provided to all participating practices: quality reporting and provider continuing medical education on patient-centered support for diabetes self-management. Patients in the HC+EM arm participated in individual counseling sessions focused on lifestyle management delivered by health coaches embedded in participating practices for a targeted total of 15 sessions. Patients in the TM+EM arm received text messages within educational, motivational, and goal-setting frames related to healthy eating, physical activity, and medication taking. Text messages were personalized and tailored according to a predefined algorithm using a semi-automated process^30,31^.

Intervention fidelity was high in all treatment arms with 100% of randomized participants receiving MODEL toolkits and 30.6% accessing recommended online resources. The fidelity of the HC approach to motivational interviewing was regularly assessed. HC+EM arm participants received a mean of 9.04 health coaching visits, and TM+EM arm participants received an average (SD) of 348 (169) text messages over 1 year of study participation.

### Outcomes and Data Collection

The primary patient-reported outcomes included three of the six subscales of the Summary of Diabetes Self-Care Activities questionnaire assessing the number of days in the last week the participants: (1) ate healthy meals, (2) participated in 30 min of physical activity, and (3) took medications as prescribed^32^. The primary outcome was measured as the mean change from baseline at 12 months. Secondary outcomes included change in (1) HbA_1c_(mean and proportion achieving HbA1c < 8%)^33^, (2) diabetes-specific quality of life using Diabetes-39^34^, (3) weight assessed as body mass index (BMI), and (4) primary care engagement using the National Health Interview Survey questions regarding delayed needed care and usual sources of care^35^. Other outcomes of interest included the Adherence to Refills and Medications Scale for Diabetes to assess medication adherence^36^, the Patient Assessment of Chronic Illness Care (PACIC) overall scale to assess quality of care from the patient’s perspective^37^, self-efficacy as assessed by the Diabetes Empowerment-Short Form (DES-SF)^38^, and systolic/diastolic blood pressure. All survey measures were assessed at baseline, 3 months, 6 months, and 12 months^26^. HbA_1c_ was assessed using EHR data as part of usual care in the 6-month period prior to MODEL Program randomization and in the 6-month window extending 3 months before and 3 months after program completion.

### Statistical Analysis

Power calculations were based on specific, planned among- and within-group contrasts. Mid-stream revision of group sizes reduced the number of randomized participants retained over the 1-year study period from 800 to 581 due to recruitment difficulties^26^. Effectiveness of improving each of the three diabetes self-care variables was defined as a positive change from baseline in any of the three arms. Based on 581 participants completing the trial, using two-sided *t*-tests within arm in the context of repeated-measures ANOVA and a type I error rate of 0.05, power exceeded 0.90 to detect a change from baseline in any of the three arms of 1.6 ± 2.2 day/week, 1.1 ± 2.4 day/week, and 0.9 ± 2.5 day/week for the three primary self-care variables, respectively^15,16,19^.

Baseline characteristics were compared among the three arms using chi-square tests for categorical variables and ANOVA for continuous variables. The outcomes at baseline, 3 months, 6 months, and 12 months were analyzed with the MIXED procedure in SAS, using repeated-measures, mixed-effects models with autoregressive covariance structures. This procedure uses maximum likelihood estimation and is robust with respect to data missing at random. Available data for all participants were included in all statistical analyses based on intention-to-treat. Urban versus suburban/rural residence was defined at ZIP Code level based on classifications proposed by Schnake-Mahl et al.^39^ Health literacy was based on a single item question “How confident are you filling out medical forms by yourself?” proposed by Chew et al.^40^and high psychosocial complexity was defined as the presence of one or more risk factors, including depression, anxiety, illegal drug use, alcoholism, or risk of homelessness. Alpha was reduced for multiplicity correction using the Holm-Bonferroni sequential method as required by PCORI methodology standards^41^.

### Role of the Funding Source

This study was supported by the Patient-Centered Outcomes Research Institute (PCORI), which advised on study design, data collection, analysis, and interpretation of the data. PCORI did not contribute to the writing of this manuscript or the decision to submit it for publication.

## RESULTS

### Trial Participants

A total of 666 patients were enrolled in the trial; 253 were assigned to the TM+EM arm, 258 to the HC+EM arm, and 155 to the EM-alone arm. The study population (Table 1) was predominantly female (67.1%), < 60 years of age (65.3%), and living in low-income areas or HPSA (90.2%) and urban areas (84.1%). Additionally, 26.9% had low health literacy, 83.0% were smart phone owners (e.g., vs. other text-capable mobile phone owners), and 39.0% had high psychosocial complexity. The three randomized groups were well balanced with respect to baseline characteristics.

A total of 588 (88.3%) of the trial participants (229 of 253 [90.5%] in the TM+EM arm, 217 of 258 [84.1%] in the HC+EM arm, and 142 of 155 [91.6%] in the EM-alone arm) completed their 12-month visits and had complete data on the primary outcomes. Patients who completed the study were generally similar in baseline characteristics to non-completers (Supplemental Table 2). A total of 349 of 588 trial participants with complete primary outcome data (59.4%) had complete secondary outcome data for HbA_1c_ from their EHR. Patients with complete EHR data were generally similar in baseline characteristics to those with complete data on primary outcomes (Supplemental Table 3).

### Primary Outcome: Diabetes Self-Care Activities

Participants in all three arms experienced significant improvements in healthy eating (Fig. 1A). On average, participants increased the number of days that they ate healthily by the equivalent of 1 day every 10 days (0.67 ± 0.19 day/week; *p* = 0.0004) in the EM-alone arm, by 1 day per 7 days (0.99 ± 0.15 day/week; *p* < 0.0001) in HC+EM, and by 1 day every 5 days (1.36 ± 0.15 day/week; (*p* < 0.0001) in TM+HC. The average improvement in healthy eating among those randomized to TM+EM was significantly greater than among those randomized to EM-alone (0.69 ± 0.24 day/week) (*p* = 0.004) (Supplemental Table 4).

Participants in all three arms also experienced significant improvements in exercise (Fig. 1B). On average, participants in the EM-alone arm increased the number of days that they exercised by the equivalent of 1 day over 8 days (0.83 ± 0.24 day/week) while those randomized to either the HC+EM or TM+EM arm increased the number of days per week that they exercised by the equivalent of about 1 day per 7 days (0.96 ± 0.19 day/week and 1.10 ± 0.18 day/week, respectively; both *p* < 0.0001) with no significant differences in improvement being detected among the arms (Supplemental Table 4).

Only TM+EM was effective in improving the average number of days participants took their diabetes medications as recommended (Fig. 1C). Average patient-reported baseline medication adherence was very high, exceeding 6 days per week in all three arms (Supplemental Table 4). TM+EM participants increased the average number of days that they took their medications as recommended by the equivalent of 1 day every 23 days (*p* = 0.0091).

### Secondary Outcomes

Participants randomized to all three arms significantly improved their HbA_1c_ from baseline to 12 months (Supplemental Table 5). Average reduction in HbA_1c_ among the 349 of 666 enrolled participants is −0.76% ± 0.12% (*p* ≤ 0.0001) and 90 of 349 participants (25.8%) with HbA_1c_ values within the window for their 12-month follow-up assessment achieved reductions below 8%. In the EM-alone arm, 21.25% (17 of 80) achieved an HbA_1c_ below 8%; in the HC+EM arm, 28.17% (40 of 142); and in the TM+EM arm, 25.98% (33 of 127). No significant differences in HbA_1c_ were detected among the three arms. Only HC+EM was effective in reducing BMI significantly from baseline to 12 months (−0.69 ± 0.27; *p* ≤ 0.0125).

Self-reported medication adherence improved significantly in all three arms (Supplemental Table 6). Self-efficacy improved among those in the HC+EM and TM+EM arms but not the EM-alone arm. Patient-reported quality of life and quality of care remained unchanged in all three arms.

### Heterogeneity of Treatment Effects

Pre-specified subpopulation analyses demonstrated that subgroups with male gender, urban residence, smartphones, low health literacy, and low psychosocial complexity experienced greater improvements in healthy eating with TM+EM than with EM alone (Fig. 2A). The male subgroup also experienced greater improvement in healthy eating with TM+EM than with HC+EM. The most pronounced effect was observed in the suburban/rural group for the exercise outcome. Additionally, TM+EM was more effective than EM alone and HC+EM in promoting exercise for suburban/rural residents (Fig. 2B). Although the confidence interval for the suburban/rural group differed significantly from the overall treatment effect, the difference was not statistically significant after adjusting for multiple comparisons.

## DISCUSSION

This large pragmatic trial is among the first to directly compare the effectiveness of diabetes self-care interventions employed in primary care settings. To our knowledge, no previous RCTs have directly compared EM, HC, and TM. Patient-expert input demonstrates that these are among the most valuable primary care interventions to support diabetes self-care from the patient perspective^23,25,42^. The MODEL Study demonstrates that these three low-cost, yet underutilized, patient-centered diabetes self-care interventions all have similar and clinically meaningful real-world effectiveness in improving self-care among African-American adults with uncontrolled diabetes. Previous RCTs have demonstrated the effectiveness of HC and TM^8,9,15,16,19–21^, but none of these trials has adequately assessed the comparative effectiveness of EM, HC, and TM in real-world settings.

The MODEL Study also found that TM+EM was significantly more effective than EM-alone in improving healthy eating and significant improvements in medication adherence were only observed in the TM+EM arm, consistent with previous studies^19,21,23^. Our qualitative results show that emphasis on daily accountability contributed to TM effectiveness^43^.

Of note, even though all three arms experienced significant improvements in HbA_1c_, significant improvements in BMI were only observed in the HC+EM arm. Our qualitative results suggest that HC intervention emphasis on healthy eating and weight loss contributed to its effectiveness^43^. Previous pilot studies also show that HC is particularly effective in promoting weight loss^9,44^. This finding is consistent with previous research indicating that person-centered and relationship-based behavioral interventions are most effective for weight loss^10,11,45^.

**Figure 3:**
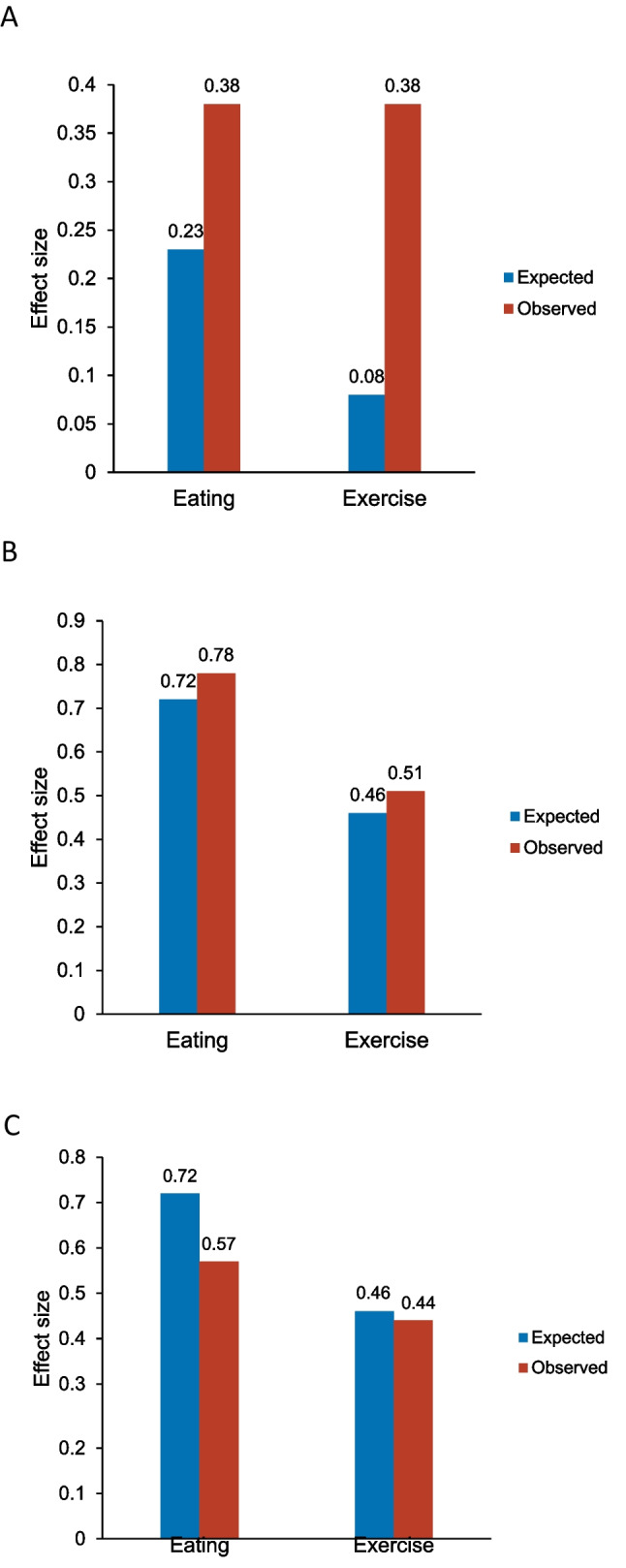
Observed and expected effectiveness of educational materials (EM) alone, health coaching (HC+EM), and text messaging (TM+EM) on patient-reported diabetes self-care activities. **A** EM-alone. **B** TM+EM. **C** HC+EM. Standardized differences were computed as the mean differences from baseline to 12 months divided by the respective standard deviations of the mean differences) based on expected differences from prior randomized clinical trials^10,11,13^ and the observed differences and respective standard deviations for healthy eating and exercise from the three arms of MODEL.

The standardized observed effectiveness of the EM-alone arm far surpassed expectations and resulted in our not finding significant differences between HC+EM and EM-alone (Fig. 3). We found that the observed effectiveness of our EM intervention was 1.65 times more effective than expected for healthy eating and 3.75 times more effective for exercise (Fig. 3A). Most previous studies of HC and TM employed usual care controls^9,15–22^; thus, when EM-alone was employed as an active comparator as required by PCORI methodology standards, its actual effects were underestimated^41^. This study demonstrates that robust patient-vetted EM is not equivalent to usual care and has greater effectiveness on diabetes self-care than expected based on previous research. Delivery of print diabetes educational materials has the potential to be a novel and potent support for diabetes self-care in medically underserved populations.

The MODEL Study has some limitations. Because we employed an active comparator (i.e., EM), the study does not assess the impact of TM or HC alone, but rather their effectiveness when combined with EM in real-world settings. Focus groups and key informant interviews revealed that participants experienced receipt of diabetes educational materials and regular meetings with research staff as support for diabetes self-care. Thus, it should not be surprising that there were only small differences among arms in intervention effectiveness. All groups received active interventions that were guided by and co-designed by the patients themselves. Recruitment from primary care resulted in reported high adherence to chronic disease medications at baseline. Also, the HC+EM arm suffered high turnover of health coaches and difficulties integrating HC into clinic workflow, likely contributing to reduced observed effectiveness of HC relative to that expected (Fig. 3C). Since patients knew they were participating in a research study, it could be argued that the MODEL Study did not truly rate real-world effectiveness, but it met the definition of a pragmatic comparative effectiveness trial and better approximated real-world conditions than many previous efficacy studies (Supplemental Table 1). In addition, since the trial required patients to complete a 2-week run-in period demonstrating responsiveness to texts and voicemail messages, it is possible that this requirement may have self-selected more motivated patients, limiting the generalizability of the findings. Furthermore, our findings apply specifically to people living in medically underserved areas and may not be generalizable to other populations.

The clinical and policy implications of this research are profound. By demonstrating that low-cost EM-alone, HC+EM, and TM+EM have clinically relevant effects on key population health outcomes, the MODEL Study supports the dissemination of these behavioral interventions in routine primary care practice. Our data provide evidence that delivery of all three of these interventions is not cost prohibitive. Estimated costs per participant for 1 year of study participation—ranging from $39 for EM-alone, $222 for the TM+EM, and $4248 for the HC+EM—are comparable to costs for other accepted interventions for this high-risk population^46^. The overall study retention of 88.3% in the MODEL Study demonstrates high demand for diabetes self-care services by the target population. Given that the HC+EM intervention was the most labor intensive, higher per participant costs should not be surprising. However, an argument can be made that HC+EM provides the highest value since this intervention arm experienced the highest patient satisfaction and was the only arm in which participants achieved significant weight loss. Furthermore, although we reported lower costs for the TM+EM intervention and other previous studies have also claimed that tailored text messaging is low cost^19–23^, implementation of the TM intervention was substantially facilitated by research personnel in the MODEL Study. In our subsequent research, we found that dissemination and implementation of TM in health systems is logistically very difficult and is associated with high fixed administrative costs^31^ that were likely underestimated in the current study. EM administrative costs may have been similarly underestimated. Yet the MODEL Study remains among the first trials to provide actionable information on implementation costs for promising alternative patient-centered behavioral interventions for improving diabetes self-care.

Improvements in HbA_1c_ and BMI associated with MODEL Study interventions should translate into improvements in diabetes complications and major cardiovascular outcomes. The average observed lowering in HbA_1c_ of –0.76% is similar to that seen with most oral antidiabetic medications. Data from the United Kingdom Prospective Diabetes Study (UKPDS) demonstrated that a HbA_1c_ reduction of only 1% was associated with a 25% decrease in microvascular disease, a 14% decrease in myocardial infarctions, and a 43% decrease in amputation or death from peripheral vascular disease^47^. Furthermore, UKPDS showed that any improvement in glycemic control was beneficial. Similarly, the Look Ahead trial demonstrated that weight loss of 10–15% in individuals with diabetes was associated with a 21% decrease in combined cardiovascular death, non-fatal acute myocardial infarction, non-fatal stroke, and hospitalization for angina, suggesting that the HC intervention may be particularly beneficial^48^. Thus, the expected cardiovascular benefits of all three low-cost MODEL interventions strongly suggest their cost-effectiveness and argue for their expanded availability.

In summary, the MODEL Study provides evidence of effectiveness for all three MODEL Study diabetes self-care interventions and suggests that people in medically underserved areas with uncontrolled diabetes would benefit from increased access to EM, HC, and TM. People in predominantly African-American medically underserved areas lack access to essential support for diabetes self-care through proven low-cost approaches^5,14,49^, and people want these services when they are delivered in patient-centered ways^23,25,42^. The MODEL Study results suggest potential for widespread application and dissemination of EM, HC, and TM in primary care settings nationwide.

## Supplementary Information

Below is the link to the electronic supplementary material.

Supplementary Material 1 (DOCX 349 KB)
