# Supplementary material for: Comparative Effectiveness of Diabetes Self-Care Interventions in African-American Adults: A Three-Arm Randomized Controlled Trial
Source: J Gen Intern Med. 2025 Oct 20;41(6):1573–83. doi: 10.1007/s11606-025-09882-z (PMC13125478; doi:10.1007/s11606-025-09882-z)
Supplement: Supplementary file 1 — Supplementary Material 1 (DOCX 349 KB) [file 11606_2025_9882_MOESM1_ESM.docx]

**Comparative Effectiveness of Diabetes Self-Care Interventions in African-American Adults — A Three-Arm Randomized Controlled Trial**

**Online-Only Supplemental Material**

Supplemental Table 1. PRagmatic Explanatory Continuum Indicator Summary 2 scores for trial domains.

Supplemental Table 2. Selection Process for Print Diabetes Education Material

Supplemental Figure 1. Patient Rating of Health Education Material

Supplemental Figure 2. Best Practice for Delivery of Low Literacy Diabetes Education Materials

Supplemental Table 3. Baseline characteristics of study completers and non-completers.

**Supplemental Table 4.** Effectiveness and comparative effectiveness of educational materials (EM) alone, health coaching (HC+EM), and text messaging (TM+EM) on three subscales of the summary of diabetes self-care questionnaire.

Supplemental Table 5. Effectiveness and comparative effectiveness of educational materials (EM) alone, health coaching (HC+EM), and text messaging (TM+EM) on secondary clinical outcomes.

Supplemental Table 6. Effectiveness and comparative effectiveness of educational materials (EM) alone, health coaching (HC+EM), and text messaging (TM+EM) on secondary patient-centered outcomes.

**Supplemental Table 1**. PRagmatic Explanatory Continuum Indicator Summary 2 (PRECIS-2) Scores for Trial Domains

|  | **Domain** | **Score** | **Rationale** |
| --- | --- | --- | --- |
| **1** | **Eligibility Criteria** | 4 | Inclusion criteria were made as broad as possible to mirror those of the larger national target population of approximately 6 million African American adults with diabetes in medically underserved areas. Many previous studies have focused on diabetes in isolation. In contrast, our study recognized that the presence of MCC in DM is the norm, and studies assessing the self-care needs of people with DM seeking broad generalizability and potential for wide-spread application, must assess self-care needs within the context of MCC. The exclusion criteria were likewise very limited to maximize generalizability, c/w a pragmatic trial. The most restrictive eligibility criteria which led us to reduce the score in this category to 4 were related to the requirement of HbA1c ≥8% and receipt of care at a participating practice. We recognize that some patients in the target population do not routinely participate in primary care. |
| **2** | **Recruitment Path** | 3 | Potentially eligible patients were identified and recruited using: (1) a comprehensive regional diabetes registry; (2) direct referral by clinic personnel or primary care providers; (3) study staff review of patient charts; (4) recruitment materials displayed in participating clinics and hospitals; and (5) community-based recruitment strategies, including health fairs, press releases, UTHSC listservs, and distribution of materials at churches and community centers. Research staff typically contacted potential participants by telephone or answered calls from interested patients who responded to marketing efforts. For some clinic referrals, clinic staff or HCPs introduced study staff to patients during regularly scheduled patient visits. Thus, although members of the target population had ready opportunity to participate, the use of research study staff for recruitment did not fully represent real world conditions. |
| **3** | **Setting** | 5 | We sought to implement the study in real-world health care settings to enhance the generalizability of our findings, consistent with the Chambless criteria for evaluating effectiveness across real-world clinical settings. Thus, the study sites were chosen to reflect the diversity of primary care and specialty practice sites in medically underserved areas nationwide. Clinics were considered to serve medically underserved areas if the residences of  more than 70% of the clinic’s patients with diabetes were located in medically underserved areas, as defined by the Health Resources & Services Administration (HRSA). The  characteristics of the patients with diabetes in the 18 participating practices in MODEL mirror those of the larger national target population of approximately 6 million African American adults with diabetes in medically underserved areas. |
| **4** | **Organization intervention** | 4 | The organization and expertise needed to implement the intervention was largely available in the participating primary care practices and health systems. The participating health systems paid for the majority of the intervention costs and the health coaches, which were the largest cost portion of the intervention, were hired and employed by the participating practices, not by the study. |
| **5** | **Flex of experimental intervention – Delivery** | 4 | High level of flexibility. We explicitly permitted patients to drop out of the intervention at any time. Educational materials were provided to all participants at regular clinic visits. The health coaching intervention was accessed in-person in the clinics where patients received regular care but was also made available by phone. The text messaging intervention was designed to be highly patient-centered and accessed through the patient’s regular cell phone without a requirement of downloading an app. But since study personnel were highly involved in the crafting and delivery of the text messages in the trial text message delivery was not fully pragmatic. |
| **6** | **Flex of experimental intervention – Adherence** | 5 | Full flexibility. We explicitly permitted patients to decrease participation or drop out of the intervention at any time. Participants were also allowed to withdraw at any time with a verbal request to study staff. |
| **7** | **Follow up** | 3 | Even though the majority of follow-up occurred in usual care settings, the intensity of measurement and follow-up of participants in the trial was significantly higher than would be expected in usual care. |
| **8** | **Outcome** | 5 | Outcomes were highly patient-centered and relevant to participants because they were selected by patients in the target population c/w PCORI methodology best practices. |
| **9** | **Analysis** | 5 | Results were analyzed based on an intention-to-treat approach. |
| **Total Score** | | 38* | |

* The PRECIS-2 has a maximum total score of 45. Thus, a score of 38 suggests a high level of pragmatism in the design and implementation of the trial (26).

**Supplemental Table 2**. Selection Process for Print Diabetes Education Material

| **Step** | **Domain** | **Detailed Description** |
| --- | --- | --- |
| **1** | **Collection of material in current use** | Collection of materials currently in use in participating practices  and additional materials recommended by healthcare professionals and certified diabetes educators. |
| **2** | **Initial material review** | Initial review of these materials by a certified diabetes recognition program director and expert health behavior researchers to eliminate materials with low relevance, understandability, visual appeal, and/or actionability. |
| **3** | **Secondary material review for reading level** | Identification and selection of all materials at a 6th-grade reading level, with clear and relevant content, and low health literacy level. |
| **4** | **Patient rating of materials** | Rating of these materials by Patient Advisory Council members from the target population on relevance, understandability, visual appeal, and actionability using a Patient Diabetes Educational Material Rating Form developed by the study team (see Supplemental, Figure 1). The form included key domains identified by the Patient Education Materials Assessment Tool. |

Supplemental Figure 1. Patient Rating of Health Education Material


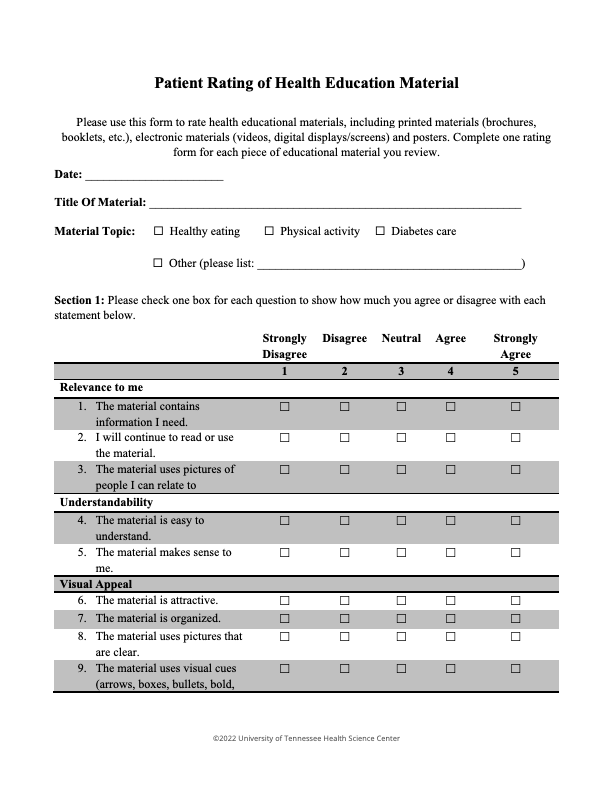


Supplemental Figure 2. Best Practice for Delivery of Low Literacy Diabetes Education Materials
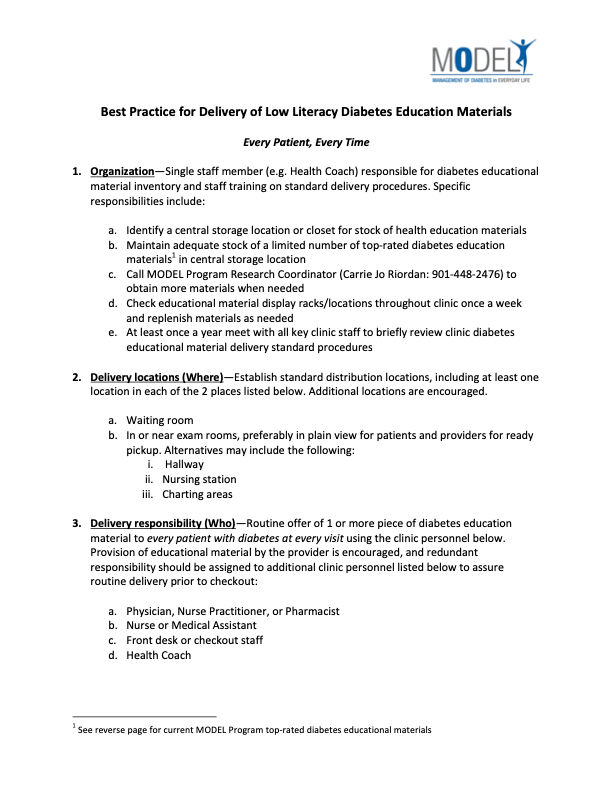

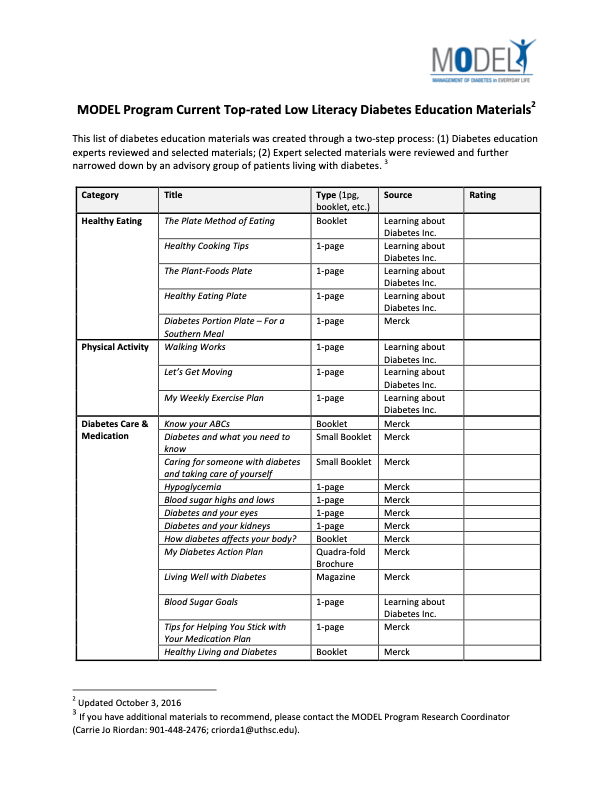


**Supplemental Table 3.** Baseline characteristics of study completers and non-completers

| **Characteristic** | **Completers***  **(N=588)** | **Non-Completers^†^**  **(N=78)** | ***P* Value^‡^** |
| --- | --- | --- | --- |
| **Age, years, mean (SD)** | 54.2 (11.5) | 53.6 (12.0) | 0.64 |
| **Age, n (%)** |  |  |  |
| < 60 years | 382 (65.0) | 53 (67.9) | 0.60 |
| ≥ 60 years | 206 (35.0) | 25 (32.1) |  |
| **Gender, n (%)** |  |  |  |
| Female | 402 (68.4) | 45 (57.7) | 0.05 |
| Male | 186 (31.6) | 33 (42.3) |  |
| **Marital status,** **n (%)** |  |  |  |
| Married or living with partner | 174 (29.6) | 22 (28.2) | 0.80 |
| Single / Separated / Divorced / Widowed | 414 (70.4) | 56 (71.8) |  |
| **Education, n (%)** |  |  |  |
| Grades 6 to 12 | 110 (18.7) | 15 (19.2) | 0.32 |
| GED / high school diploma | 152 (25.9) | 26 (33.3) |  |
| Some college / associate / bachelors / graduate degree / other, n (%) | 326 (55.4) | 37 (47.4) |  |
| **Health literacy**^†^**, n (%)** |  |  |  |
| Low health literacy | 156 (26.5) | 23 (29.5) | 0.6 |
| High health literacy | 432 (73.5) | 55 (70.5) |  |
| **Urban/Suburban-Rural status**^†^**, n (%)** |  |  |  |
| Residence in urban areas | 493 (83.8) | 66 (84.6) | 0.86 |
| Residence in suburban / rural areas | 95 (16.2) | 12 (15.4) |  |
| **Comorbidities**^‡^**, n (%)** |  |  |  |
| Hypertension | 537 (91.3) | 70 (89.7) | 0.64 |
| Asthma | 89 (15.1) | 11 (14.1) | 0.81 |
| Hyperlipidemia | 445 (75.7) | 64 (82.1) | 0.21 |
| Arthritis | 232 (39.5) | 37 (47.4) | 0.18 |
| Cancer | 25 (4.3) | 8 (10.3) | 0.02 |
| Stroke | 60 (10.2) | 13 (16.7) | 0.09 |
| Chronic obstructive pulmonary disease | 26 (4.4) | 9 (11.5) | 0.008 |
| Congestive heart failure | 59 (10.0) | 13 (16.7) | 0.08 |
| Coronary artery disease | 37 (6.3) | 11 (14.1) | 0.01 |
| Cardiac arrhythmias | 44 (7.5) | 10 (12.8) | 0.10 |
| Depression | 140 (23.8) | 21 (26.9) | 0.55 |
| Chronic kidney disease | 56 (9.5) | 14 (17.9) | 0.02 |
| Osteoporosis | 22 (3.7) | 2 (2.5) | -- |
| **Number of comorbidities, n (%)** |  |  |  |
| 1 comorbidity | 81 (13.8) | 8 (10.3) | 0.07 |
| 2-3 comorbidities | 319 (54.2) | 35 (44.9) |  |
| ≥ 4 comorbidities | 188 (32.0) | 35 (44.8) |  |
| **Smart phone ownership**^§^**, n (%)** |  |  |  |
| Patients with no smart phone | 102 (17.4) | 11 (14.3) | 0.50 |
| Patients with smart phone | 486 (82.6) | 66 (85.7) |  |
| **Psychosocial complexity**^∥^**, n (%)** |  |  |  |
| Low psychosocial complexity | 373 (63.4) | 47 (60.3) | 0.58 |
| High psychosocial complexity | 215 (36.6) | 31 (39.7) |  |
| **Medical complexity**^¶^**, n (%)** |  |  |  |
| No | 19 (4.1) | 2 (3.2) | -- |
| Yes | 441 (95.9) | 60 (96.8) |  |

* Study participants who completed their 12-month visit

^†^ Study participants who did not complete their 12-month visit

^‡^ Significance level is p<0.05

^§^ Urban vs. suburban and rural categories are based on Schanke-Mahl classification

^∥^ Each chronic condition was a binary variable, 1 indicating presence of the chronic condition and 0 indicating absence of the chronic condition. Chi-square tests were conducted to compare patients who had a chronic condition vs. those who did not.

^¶^ Missing data for one patient

** High psychosocial complexity is defined as either a positive screen for depression (using PHQ-9), anxiety (using GAD-7), substance abuse (using the NIDA Quick Screen and AUDIT), or housing instability (using the Homelessness Screening Clinical Reminder).

†† High medical complexity is defined according to Camden Coalition and SafeMed “high-utilizer” criteria of - two or more hospital admissions OR one inpatient admission and two or more prior emergency visits in the last 6 months prior to enrollment in the study. Of 666 participants, hospitalization and emergency department visit data were available for 522 patients.

Supplemental Table 4. Effectiveness and comparative effectiveness of educational materials (EM) alone, health coaching (HC+EM), and text messaging (TM+EM) on three subscales of the summary of diabetes self-care questionnaire*

| **Scale/visit** | (EM alone,  **n = 157†)** | (HC+EM,  **n = 259‡)** | (TM+EM,  **n = 256§)** | **HC+EM vs EM alone^a^** | TM+EM vs  **EM alone^a^** | **TM+EM vs HC+EM^a^** |
| --- | --- | --- | --- | --- | --- | --- |
| **General Diet,^∥^ mean (SE), d** |  |  |  |  |  |  |
| Baseline | 3.70 (0.14) | 3.42 (0.11) | 3.20 (0.11) | 0.07 to 0.63 | –0.16 to 0.85 | 0.08 to 0.53 |
| 3 mo | 4.25 (0.14) | 4.36 (0.11) | 4.33 (0.11) | 0.47 to 0.25 | 0.44 to 0.28 | 0.28 to 0.34 |
| 6 mo | 4.32 (0.14) | 4.52 (0.12) | 4.55 (0.11) | 0.57 to 0.16 | 0.60 to 0.12 | 0.35 to 0.28 |
| 12 mo | 4.37 (0.15) | 4.41 (0.12) | 4.55 (0.11) | 0.41 to 0.32 | 0.55 to 0.18 | 0.46 to 0.18 |
| Estimated D* | 0.67 (0.19)^¶^ | 0.99 (0.15)^¶^ | 1.36 (0.15)^¶^ | 0.33 (0.24) | 0.69 (0.24)^¶^ | 0.36 (0.21) |
| 95% CI | 0.30 to 1.04 | 0.70 to 1.28 | 1.07 to 1.64 | –0.14 to 0.79 | 0.22 to 1.16 | –0.05 to 0.77 |
| **Exercise,** mean (SE), d** |  |  |  |  |  |  |
| Baseline | 2.79 (0.17) | 2.44 (0.13) | 2.33 (0.14) | 0.08 to 0.78 | –0.03 to 0.90 | 0.27 to 0.49 |
| 3 mo | 3.64 (0.18) | 3.16 (0.14) | 3.42 (0.14) | –0.03 to 0.93 | 0.22 to 0.67 | 0.65 to 0.13 |
| 6 mo | 3.46 (0.18) | 3.40 (0.14) | 3.20 (0.14) | 0.40 to 0.51 | 0.19 to 0.71 | 0.19 to 0.60 |
| 12 mo | 3.63 (0.18) | 3.40 (0.15) | 3.43 (0.14) | 0.23 to 0.68 | 0.25 to 0.65 | 0.42 to 0.38 |
| Estimated D* | 0.83 (0.24)^¶^ | 0.96 (0.19)^¶^ | 1.10 (0.18)^¶^ | 0.13 (0.30) | 0.26 (0.30) | 0.13 (0.26) |
| 95% CI | 0.37 to 1.30 | 0.60 to 1.33 | 0.73 to 1.46 | –0.46 to 0.72 | –0.33 to 0.85 | –0.38 to 0.65 |
| **Medication Adherence,^††^ mean (SE), d** |  |  |  |  |  |  |
| Baseline | 6.40 (0.11) | 6.39 (0.08) | 6.24 (0.09) | 0.26 to 0.28 | 0.11 to 0.43 | 0.08 to 0.39 |
| 3 mo | 6.55 (0.11) | 6.41 (0.09) | 6.48 (0.09) | 0.14 to 0.42 | 0.20 to 0.35 | 0.31 to 0.18 |
| 6 mo | 6.63 (0.11) | 6.50 (0.09) | 6.42 (0.09) | 0.16 to 0.41 | 0.07 to 0.49 | 0.16 to 0.33 |
| 12 mo | 6.58 (0.11) | 6.51 (0.09) | 6.54 (0.09) | 0.22 to 0.35 | 0.24 to 0.32 | 0.27 to 0.23 |
| Estimated D* | 0.18 (0.15) | 0.12 (0.12) | 0.30 (0.11) | –0.06 (0.19) | 0.12 (0.19) | 0.18 (0.16) |
| 95% Confidence Interval | -0.11 to 0.47 | -0.10 to 0.35 | 0.07 to 0.53 | –0.42 to 0.31 | –0.25 to 0.49 | –0.14 to 0.50 |

EM = educational materials; HC = health coaching; s_pooled_ = pooled residual SD; TM = text messaging.

^a^ Estimate of the difference between baseline and 12-mo changes by specified group.

* Effectiveness is the estimate and SE of the difference between the mean at the 12-month assessment and the mean at the baseline assessment for each treatment group. Comparative effectiveness is the estimate and SE of the difference between the effectiveness of the 2 specified groups.

^†^ For the EM-alone group, 155 participants completed the baseline visit; 142 completed the 12-month follow-up visit.

^‡^ For the HC + EM group, 258 participants completed the baseline visit; 217 completed the 12-month follow-up visit.

^§^ For the TM + EM group, 253 participants completed the baseline visit; 229 completed the 12-month follow-up visit.

^∥^ *df* = 1776; s_pooled_ = 1.743. General Diet subscale: Average of the number of days in the past 7 days that participants followed a healthy eating plan and the average number of days per week over the past month that participants followed their eating plans. Least squares mean (SE) for each treatment group at each assessment time; estimate, SE, and 95% CI of the effectiveness of each group; the 95% CIs of the D between pairs of groups at each visit; and the estimate, SE, and 95% CI of the D in effectiveness between pairs of groups. Of a total of 2452 observations, 1 was omitted because of missing data.

^¶^ *P* < .0042 based on the Holm-Bonferroni sequential procedure for multiplicity correction for 18 contrasts.

** *df* = 1775; s_pooled_ = 2.174. Exercise subscale: Average of the number of days in the past 7 days that participants engaged in at least 30 minutes of continuous physical activity and the average number of days in the past 7 days that participants engaged in a specific exercise session other than around the house or as part of their work. Least squares mean (SE) for each treatment group at each assessment time; estimate, SE, and 95% CI of the effectiveness of each group; the 95% CIs of the D between pairs of groups at each visit and the estimate, SE, and 95% CI of the D in effectiveness between pairs of groups. Of 2452 observations, 2 were omitted because of missing data.

^††^ *df* = 1776; s_pooled_ = 1.356. Medication Adherence subscale: Average of the number of days in the past 7 days that participants took their recommended diabetes medication. Least squares mean (SE) for each treatment group at each assessment time; estimate, SE, and 95% CI of the effectiveness of each group; the 95% CIs of the D between pairs of groups at each visit and the estimate, SE, and 95% CI of the D in effectiveness between pairs of groups. Of 2452 observations, 1 was omitted because of missing data.

Supplemental Table 5. Effectiveness^a^ and comparative effectiveness^a^ of educational materials (EM) alone, health coaching (HC+EM), and text messaging (TM+EM) on secondary clinical outcomes

| **Variable/estimate** | (EM alone, n = 157*) | (HC+EM,  **n = 259^†^)** | (TM+EM,  **n = 256^‡^)** | **HC+EM vs EM alone^b^** | TM+EM vs  **EM alone^b^** | **TM+EM vs HC+EM^b^** |
| --- | --- | --- | --- | --- | --- | --- |
| **HbA_1C_,** ^§^ **mean (SE), %** |  |  |  |  |  |  |
| Baseline | 10.35 (0.23) | 10.10 (0.17) | 10.35 (0.18) | – | – | – |
| Estimated D^∥^ | ‒0.69 (0.25) ^¶^ | ‒0.87 (0.09) ^¶^ | ‒0.68 (0.20) ^¶^ | –0.18 (0.31) | 0.005 (0.31) | 0.18 (0.27) |
| 95% CI | ‒1.17 to ‒0.20 | ‒1.23 to ‒0.50 | ‒1.07 to ‒0.30 | ‒0.78 to 0.43 | ‒0.61 to 0.62 | ‒0.34 to 0.71 |
| **BMI,** ** **mean (SE)** |  |  |  |  |  |  |
| Baseline | 35.95 (1.18) | 35.97 (0.87) | 35.37 (0.88) | – | – | – |
| Estimated D ^∥^ | ‒0.10 (0.37) | ‒0.69 (0.27) ^¶^ | ‒0.05 (0.28) | –0.59 (0.46) | 0.04 (0.46) | 0.63 (0.39) |
| 95% CI | ‒0.82 to 0.63 | ‒1.21 to ‒0.15 | ‒0.60 to 0.49 | ‒1.49 to 0.31 | ‒0.86 to 0.95 | ‒0.13 to 1.39 |
| **Systolic blood pressure,** ^††^ **mean (SE), mm Hg** |  |  |  |  |  |  |
| Baseline | 137.38 (2.63) | 137.09 (2.03) | 132.25 (2.07) | – | – | – |
| Estimated D^∥^ | ‒1.79 (2.07) | ‒1.06 (2.08) | 2.68 (2.13) | 0.73 (3.41) | 4.47 (3.44) | 3.74 (2.98) |
| 95% CI | ‒7.12 to 3.53 | ‒5.16 to 3.04 | ‒1.52 to 6.88 | ‒5.99 to 7.45 | ‒2.31 to 11.25 | ‒2.13 to 9.61 |
| **Diastolic blood pressure,** ^‡‡^ **mean (SE), mm Hg** |  |  |  |  |  |  |
| Baseline | 83.06 (1.38) | 82.57 (1.08) | 82.52 (1.09) | – | – | – |
| Estimated D∥ | ‒1.19 (1.44) | ‒0.26 (1.12) | ‒0.47 (1.14) | 0.93 (1.88) | 0.73 (1.84) | –0.21 (1.60) |
| 95% CI | ‒4.03 to 1.65 | ‒2.47 to 1.95 | ‒2.71 to 1.78 | ‒2.67 to 4.53 | ‒2.89 to 4.34 | ‒3.36 to 2.94 |

BMI = body mass index; EM = educational materials; HbA_1c_ = hemoglobin A_1c_; HC = health coaching; MODEL = Management of Diabetes in Everyday Life; s_pooled_ = pooled residual SD; TM = text messaging.

^a^ Effectiveness is the estimate and SE of the difference between the mean at the 12-month assessment and the mean at the baseline assessment for each treatment group. Comparative effectiveness is the estimate and SE of the difference between the effectiveness of the 2 specified groups.

^b^ Estimate of the difference between baseline and 12-mo changes by specified group.

* For the EM-alone group, 155 participants completed the baseline visit, and 142 completed the 12-month follow-up visit.

^†^ For the HC + EM group, 258 participants completed the baseline visit, and 217 completed the 12-month follow-up visit.

^‡^ For the TM + EM group, 253 participants completed the baseline visit, and 229 completed the 12-month follow-up visit.

^§^ *df* = 346; s_pooled_ = 2.062. Sample sizes for HbA_1c_: n_EM alone_ = 80%; n_HC + EM_ = 142%; and n_TM + EM_ = 127%. The 12-month data include values from some participants who were lost to follow-up to MODEL but had HbA_1c_ values recorded in the registry. Of 1254 possible observations, those for 239 participants were missing at 12 months.

^∥^ Effectiveness is the estimated D between the least squares mean at the 12-month assessment and the least squares mean at the baseline assessment for each treatment group. Comparative effectiveness is the estimate of the D between specified groups of the changes over 12 months from baseline.

^¶^ *P* ≤ .0125 based on the Holm-Bonferroni sequential procedure for multiplicity correction for 6 contrasts per variable.

** *df* = 265; s_pooled_ = 8.970. Sample sizes for BMI: n_EC alone_ = 58; n_HC + EC_ = 107; and n_TM + EC_ = 103. The 12-month data include values from some participants who were lost to follow-up to MODEL but had BMI values recorded in the registry. Of 1254 possible observations, those for 320 participants were missing at 1 or both assessments.

^††^ *df* = 267; s_pooled_ = 20.871. Sample sizes for systolic blood pressure: n_EC alone_ = 63 mm Hg; n_HC + EC_ = 107 mm Hg; and n_TM + EC_ = 102 mm Hg. The 12-month data include values from some participants who were lost to follow-up to MODEL but had systolic blood pressure values recorded in the registry. Of 1254 possible observations, those for 316 participants were missing at 1 or both assessments.

^‡‡^ *df* = 265; s_pooled_ = 10.985. Sample sizes for diastolic blood pressure: n_EC alone_ = 63 mm Hg; n_HC + EC_ = 104 mm Hg; and n_TM + EC_ = 101 mm Hg. The 12-month data include values from some participants who were lost to follow-up to MODEL but had diastolic blood pressure values recorded in the registry. Of 1254 possible observations, those for 320 participants were missing at 1 or both assessments.

Supplemental Table 6. Effectiveness^a^ and comparative effectiveness^a^ of educational materials (EM) alone, health coaching (HC+EM), and text messaging (TM+EM) on secondary patient-centered outcomes

| **Instrument/estimate** | **(EM alone, n = 157*)** | (HC+EM,  **n = 259^†^)** | (TM+EM,  **n = 256^‡^)** | **HC+EM vs EM alone^b^** | TM+EM vs  **EM alone^b^** | **TM+EM vs HC+EM^b^** |
| --- | --- | --- | --- | --- | --- | --- |
| **ARMS-D, ^§^ mean (SE), points** |  |  |  |  |  |  |
| Baseline | 18.28 (0.36) | 18.32 (0.28) | 18.77 (0.29) | – | – | – |
| Estimated D ^∥^ | ‒1.48 (0.45)^¶^ | ‒1.39 (0.35)^¶^ | ‒2.16 (0.35)^¶^ | 0.09 (0.57) | –0.68 (0.57) | –0.77 (0.49) |
| 95% CI | ‒2.36 to ‒0.60 | ‒2.07 to ‒0.71 | ‒2.84 to ‒1.49 | 1.02 to 1.20 | –1.79 to 0.42 | –1.73 to 0.19 |
| **DES-SF, ** mean (SE), points** |  |  |  |  |  |  |
| Baseline | 3.83 (0.07) | 3.85 (0.06) | 3.72 (0.06) | – | – | – |
| Estimated D ^∥^ | 0.14 (0.08) | 0.27 (0.07)^¶^ | 0.35 (0.07)^¶^ | 0.13 (0.11) | 0.20 (0.11) | 0.08 (0.09) |
| 95% CI | ‒0.02 to 0.31 | 0.14 to 0.40 | 0.22 to 0.48 | –0.09 to 0.34 | –0.01 to 0.41 | –0.11 to 0.26 |
| **Diabetes-39, ^††^ mean (SE), points** |  |  |  |  |  |  |
| Baseline | 4.85 (0.12) | 4.97 (0.09) | 4.88 (0.09) | – | – | – |
| Estimated D ^∥^ | 0.38 (0.16) | 0.28 (0.13) | 0.40 (0.13) | –0.10 (0.21) | 0.02 (0.21) | 0.12 (0.18) |
| 95% CI | 0.06 to 0.70 | 0.03 to 0.53 | 0.16 to 0.65 | –0.50 to 0.31 | –0.38 to 0.42 | –0.23 to 0.47 |
| **PACIC, ^‡‡^ mean (SE), points** |  |  |  |  |  |  |
| Baseline | 3.22 (0.09) | 3.11 (0.07) | 2.94 (0.07) | – | – | – |
| Estimated D ^∥^ | ‒0.04 (0.09) | 0.17 (0.07) | 0.19 (0.07) | 0.21 (0.11) | 0.23 (0.11) | 0.02 (0.10) |
| 95% CI | ‒0.21 to 0.13 | 0.03 to 0.31 | 0.06 to 0.33 | –0.10 to 0.43 | 0.01 to 0.45 | –0.17 to 0.21 |

ARMS-D = Adherence to Refills and Medications Scale for Diabetes; DES‑SF = Diabetes Empowerment Scale–Short Form; EM = educational materials; HC = health coaching; PACIC = Patient Assessment of Chronic Illness; QOL = quality of life; s_pooled_ = pooled residual SD; TM = text messaging.

^a^ Effectiveness is the estimate and SE of the difference between the mean at the 12-month assessment and the mean at the baseline assessment for each treatment group. Comparative effectiveness is the estimate and SE of the difference between the effectiveness of the 2 specified groups.

^b^ Estimate of the difference between baseline and 12-mo changes by specified group.

* For the EM alone group, 155 participants completed the baseline visit, and 142 completed the 12-month follow-up visit.

^†^ For the HC + EM group, 258 participants completed the baseline visit, and 217 completed the 12-month follow-up visit.

^‡^ For the TM + EM group, 253 participants completed the baseline visit, and 229 completed the 12-month follow-up visit.

^§^ ARMS-D (range, 11-44): *df* = 1770; s_pooled_ = 4.554. The ARMS-D was used to assess medication adherence and refill adherence. A decrease indicates improvement. Of 2452 observations, 7 were omitted because of missing data.

^∥^ Effectiveness is the estimated D between the least squares mean at the 12-month assessment and the least squares mean at the baseline assessment for each treatment group. Comparative effectiveness is the estimate of the D between specified groups of the changes over 12 months from baseline.

^¶^ *P* < .002 based on the Holm-Bonferroni sequential procedure for multiplicity correction for 24 contrasts.

** DES-SF (range, 1-5): *df* = 575; s_pooled_ = 0.906. The DES-SF was used to assess self-efficacy. Of 1254 observations, 1 were omitted because of missing data.

^††^ Diabetes-39 (range, 1-7): *df* = 1751; s_pooled_ = 1.461. The Diabetes-39 Quality of Life subscale was used to assess diabetes-specific QOL. Of 2452 observations, 26 were omitted because of missing data.

^‡‡^ PACIC (range 1-5): *df* = 583; s_pooled_ = 1.078. The PACIC overall scale was used to assess quality of care from the patient’s perspective. Of 1254 observations, 2 were omitted because of missing data.

**References**

26. Loudon K, Treweek S, Sullivan F, Donnan P, Thorpe KE, Zwarenstein M**.** The PRECIS-2 tool: designing trials that are fit for purpose. BMJ. 2015;350:h2147.
